# The cortical activation pattern by a rehabilitation robotic hand: a functional NIRS study

**DOI:** 10.3389/fnhum.2014.00049

**Published:** 2014-02-06

**Authors:** Pyung-Hun Chang, Seung-Hee Lee, Gwang Min Gu, Seung-Hyun Lee, Sang-Hyun Jin, Sang Seok Yeo, Jeong Pyo Seo, Sung Ho Jang

**Affiliations:** ^1^Department of Robotics Engineering, Graduate School, Daegu Gyeongbuk Institute of Science and TechnologyTaegu, South Korea; ^2^Department of Mechanical Engineering, Graduate School, Korea Advance Institute of Science and TechnologyTaegu, South Korea; ^3^Robotics Research Division, Daegu Gyeongbuk Institute of Science and TechnologyTaegu, South Korea; ^4^Department of Physical Medicine and Rehabilitation, College of Medicine, Yeungnam UniversityTaegu, South Korea

**Keywords:** functional NIRS, robot, cortical activation, brain plasticity, rehabilitation

## Abstract

**Introduction:** Clarification of the relationship between external stimuli and brain response has been an important topic in neuroscience and brain rehabilitation. In the current study, using functional near infrared spectroscopy (fNIRS), we attempted to investigate cortical activation patterns generated during execution of a rehabilitation robotic hand.

**Methods:** Ten normal subjects were recruited for this study. Passive movements of the right fingers were performed using a rehabilitation robotic hand at a frequency of 0.5 Hz. We measured values of oxy-hemoglobin (HbO), deoxy-hemoglobin (HbR) and total-hemoglobin (HbT) in five regions of interest: the primary sensory-motor cortex (SM1), hand somatotopy of the contralateral SM1, supplementary motor area (SMA), premotor cortex (PMC), and prefrontal cortex (PFC).

**Results:** HbO and HbT values indicated significant activation in the left SM1, left SMA, left PMC, and left PFC during execution of the rehabilitation robotic hand (uncorrected, *p* < 0.01). By contrast, HbR value indicated significant activation only in the hand somatotopic area of the left SM1 (uncorrected, *p* < 0.01).

**Conclusions:** Our results appear to indicate that execution of the rehabilitation robotic hand could induce cortical activation.

## Introduction

In recent decades, many rehabilitation robots have been developed for patients with brain injury (Aisen et al., 1997; Krebs et al., 1998; Volpe et al., 2000; Lum et al., 2002, 2006; Kahn et al., 2006; Masiero et al., 2007; Kwakkel et al., 2008; Rabadi et al., 2008; Housman et al., 2009; Lo et al., 2010; Conroy et al., 2011; Lapitskaya et al., 2011). These rehabilitation robots have been designed to aid in improvement or to assist with functional activity of patients with brain injury. The basic principle of brain rehabilitation is based on manipulation of external stimuli, which can induce activation of the cerebral cortex (Kaplan, 1988). Therefore, clarification of the relationship between external stimuli and brain response has been an important topic in neuroscience and brain rehabilitation. Likewise, regarding use of rehabilitation robots for patients with brain injury, elucidation of brain response by execution of rehabilitation robots would be important, however, little is known about cortical activation patterns induced by proprioceptive inputs during execution of rehabilitation robots (Blicher and Nielsen, 2009; Kamibayashi et al., 2009; Li et al., 2012).

Several functional neuroimaging techniques, including functional MRI (fMRI), Positron Emission Tomography, and functional near infrared spectroscopy (fNIRS) are available for use in brain activation studies by external stimuli (Frahm et al., 1993; Miyai et al., 2001; Perrey, 2008; LaPointe et al., 2009; Mihara et al., 2010; Kim et al., 2011; Leff et al., 2011; Gagnon et al., 2012). Among these techniques, fMRI, which can be employed repeatedly, produces no ionizing radiation, and shows high spatial resolution, has been used most frequently (Frahm et al., 1993). However, it is sensitive to artifact resulting from motion and metallic materials. Robots are usually made of metal, and rehabilitation robots can perform large movements. Therefore, fMRI might not be appropriate for use in brain activation study of rehabilitation robots although some studies have reported cortical effects by magnetic resonance-compatible rehabilitation robots (Tsekos et al., 2007; Astrakas et al., 2012). By contrast, less sensitivity of fNIRS to motion artifact and metal material has been demonstrated; therefore, fNIRS could be more appropriate for use in research on brain activation study of rehabilitation robots (Arenth et al., 2007; Irani et al., 2007; Perrey, 2008; Mihara et al., 2010; Leff et al., 2011). In addition, compared with other functional neuroimaging techniques, the fNIRS has the advantage of applicability in more realistic day-to-day rehabilitation settings.

Many previous studies have reported on cortical activation by passive movements of a joint (Reddy et al., 2001; Radovanovic et al., 2002; Chang et al., 2009; Szameitat et al., 2012). According to these previous studies, passive movements induced cortical activation mainly in the primary sensorimotor cortex (SM1), and premotor cortex (PMC) and supplementary motor area (SMA) are also involved in processing of somatosensory input by passive movements (Reddy et al., 2001; Radovanovic et al., 2002; Chang et al., 2009). In addition, it has been shown that cortical activation patterns during passive movements were similar to that of active movements (Szameitat et al., 2012). In this study, we developed a rehabilitation robotic hand and hypothesized that performance of passive movements by our rehabilitation robotic hand can induce the proper amount of somatosensory stimulation to induce activation of the cerebral cortex, particularly the contralateral SM1, PMC and SMA.

In the current study, using fNIRS we attempted to investigate cortical activation patterns generated during execution of a rehabilitation robotic hand.

## Subjects and methods

### Subjects

Ten healthy right-handed subjects (6 males, 4 females; mean age 27.8 ± 2.5 years, range 24–32) with no history of neurological, physical, or psychiatric illness were recruited for this study. All subjects were asked to complete a questionnaire in order to confirm that they had no history of neurological, physical, or psychiatric illness. They understood the purpose of the study and provided written, informed consent prior to participation. The study protocol was approved by our Institutional Review Board.

### The rehabilitation robotic hand

**Figure 1:**
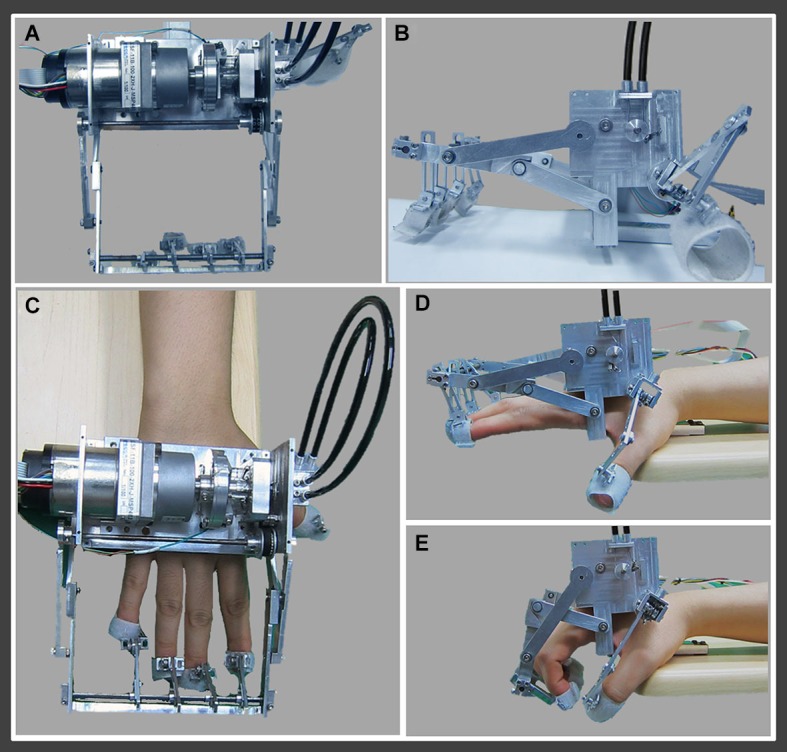
**Rehabilitation robotic hand. (A)** Superior view of the rehabilitation robotic hand, **(B)** lateral view of the rehabilitation robotic hand, **(C)** fixed state of the right hand to the rehabilitation robotic hand, **(D)** finger extension (second to fifth fingers) and thumb abduction state by the rehabilitation robotic hand, **(E)** finger flexion (second to fifth fingers) and thumb adduction state by the rehabilitation robotic hand.

The rehabilitation robotic hand consists of two moving parts for four fingers (from second to fifth finger) and thumb (Figures 1A, B). The moving part for four fingers consists of a finger holder, four bar linkage, one actuator, and a force-torque sensor system, which is driven by a timing belt (Figure 1C). The finger holders are made with Velcro straps that allow the human’s hand to follow the end point trajectory of the robot (Figure 1D). The grasping motion of the human’s hand can be realized using four bar linkages, which are designed by imitating the trajectory of real grasping motion (Figure 1E). The moving part for the thumb consists of two wires, two pulleys, and a tension adjuster. It is directly connected to the moving part for four fingers by the cable driven system. It uses only 1 degree of freedom (DOF) for grasping motion. When the motor rotates positive 90°, it performs grasping motion, and rotation of negative 90° results in performance of extension motion. Continuous action of the motor results in realization of repetitive flexion-extension motion. For real time control, using the linux distros Fedora 10 with linux kernel ver. 2.6.24 and Realtime Hardware Abstraction Layer (RTAI) ver. 3.7.1 systems, using an encoder and a Sensoray s626 board, we realized real time sensing control. For more precise control, we used time delay control. As a result, the rehabilitation robotic hand showed a position error of 0.1 ~ 1°.

All subjects were asked to sit comfortably on a chair in an upright position during conduct of the experiment. They were instructed to relax their hands maximally and not to move the hand voluntarily or imagine the movements during performance of passive movements, which were executed by the robotic hand. The subject’s right hand was fixed at the rehabilitation robotic hand. Using a block paradigm design (three cycles; resting (20 s)-movement by the robot (20 s)-resting (20 s)-movement by the robot (20 s)-resting (20 s)-movement by the robot (20 s)) at a frequency of 0.5 Hz, flexion-extension movements of the right fingers (from second to fifth) and abduction-adduction of the right thumb were performed by the rehabilitation robotic hand. During performance of passive hand movements, one experimenter confirmed that there had been no movement of the shoulder, elbow and wrist, and another experimenter observed changes in cortical activities using the monitor screen of the fNIRS system on a real-time basis (Yu et al., 2011; Dinomais et al., 2013).

### Functional near infrared spectroscopy (NIRS)

The fNIRS system (FOIRE-3000; Shimadzu, Kyoto, Japan), with continuous wave laser diodes with wavelengths of 780, 805, and 830 nm, was used for recording of cortical activity at a sampling rate of 10 Hz; we employed a 49-channel system with 30 optodes (15 light sources and 15 detectors). Based on the modified Beer–Lambert law, we acquired values for oxy-hemoglobin (HbO), deoxy-hemoglobin (HbR), and total-hemoglobin (HbT: mmol) following changes in levels of cortical concentration (Cope and Delpy, 1988). The international 10/20 system, with cranial vertex (Cz) located beneath the 18th channel, between the fourth light source and the seventh detector, was used for positioning of optodes; locations of the nasion, left ear, and right ear were identified in each subject. A stand-alone application was used for spatial registration of the 49 acquired channels on the Montreal Neurological Institute (MNI) brain based on locations of the nasion, left ear, and right ear, and the 18th channel on the Cz (Ye et al., 2009).

The software package NIRS-SPM^1^ implemented in the MATLAB environment (The Mathworks, USA) was used in analysis of fNIRS data. Gaussian smoothing with a full width at a half maximum (FWHM) of 2 s was applied to correction of noise from the fNIRS system (Ye et al., 2009). The wavelet minimum description length (MDL) based detrending algorithm was used for correction of signal distortion due to breathing or movement of the subject and general linear model (GLM) analysis with a canonical hemodynamic response curve was then performed in order to model the hypothesized HbO response under the experimental condition (Ye et al., 2009). Statistical parametric mapping (SPM) *t*-statistic maps were computed for group analysis, and, for stricter analysis, HbO, HbR, and HbT were considered significant at an uncorrected threshold of *p* < 0.01 (Ye et al., 2009).

**Figure 2:**
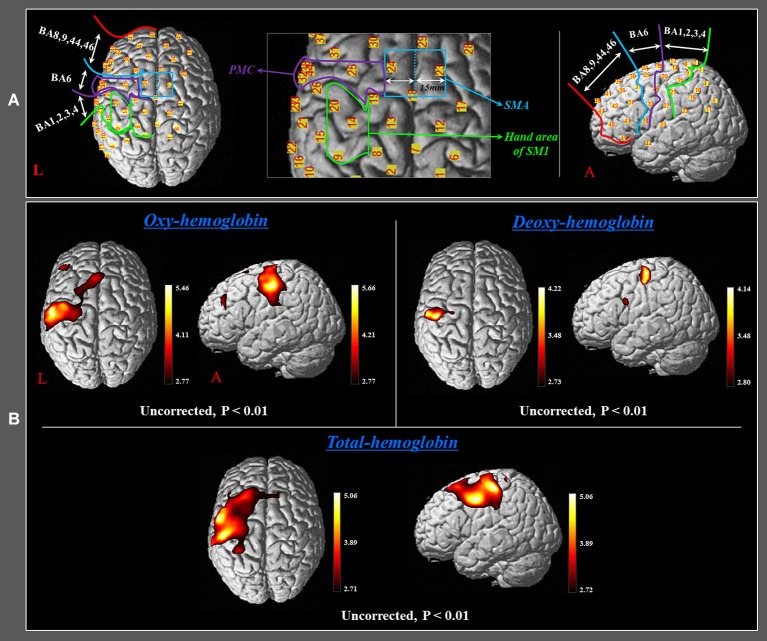
**(A)** Four regions of interest based on the Brodmann area (BA) and anatomical location of areas of the brain. The primary sensory-motor cortex (SM1): BA 1, 2, 3, and 4; premotor cortex (PMC): BA 6 (BA 6, except for the SMA); supplementary motor area (SMA) (anterior boundary: vertical line to the anterior commissure, posterior boundary: anterior margin of M1, medial boundary: midline between the right and left hemispheres, lateral boundary: the line 15 mm lateral from the midline between the right and left hemispheres); prefrontal cortex (PFC): BA 8, 9, 44, 45, and 46. **(B)** Group-average activation map of HbO, HbR, and HbT during performance of passive movements of the right fingers, which were executed by the rehabilitation robotic hand using NIRS-SPM (uncorrected, *p* < 0.01).

In order to investigate the cortical changing aspects of HbO, HbR and HbT during performance of finger movements, which were executed by the rehabilitation robotic hand, we selected five regions of interest (ROI) based on the Brodmann area (BA) and anatomical locations of brain areas: SM1 (BA 1, 2, 3, and 4), the hand somatotopic area of the SM1 (medial boundary: medial margin of the precentral knob, lateral boundary: lateral margin of the precentral knob), PMC (BA 6, except for the SMA), SMA (anterior boundary: vertical line to the anterior commissure, posterior boundary: anterior margin of primary motor cortex (M1), medial boundary: midline between the right and left hemispheres, lateral boundary: the line 15 mm lateral from the midline between the right and left hemispheres), and the prefrontal cortex (PFC) (BA 8, 9, 44, 46) (Figure 2A; Afifi and Bergman, 2005; Mayka et al., 2006).

## Results

**Figure 3:**
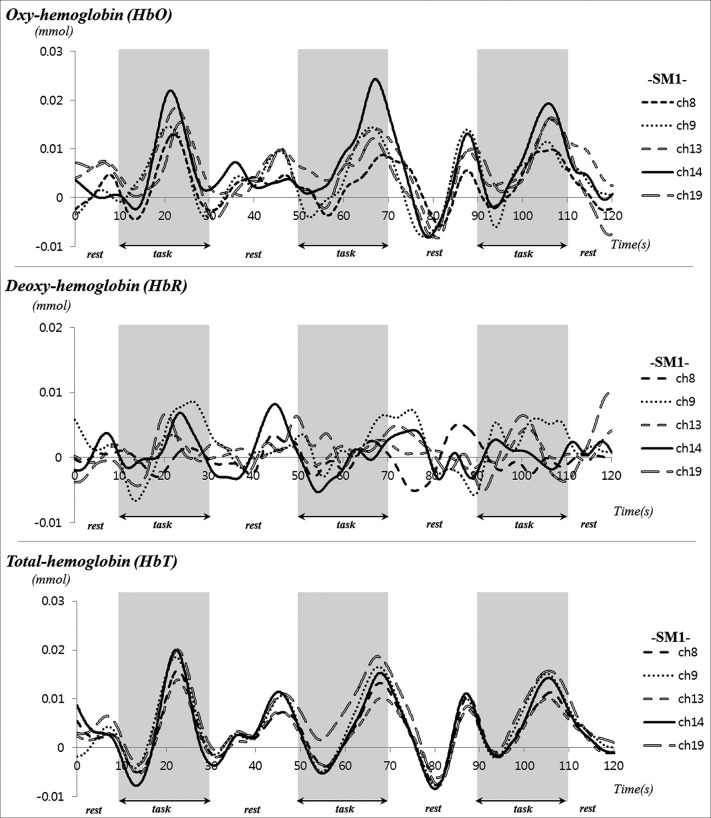
**Time course of hemodynamic responses for oxy-hemoglobin (HbO) deoxy-hemoglobin (HbR) and total-hemoglobin (HbT) in region of interest on the sensory motor cortex during performance of passive movements of the right fingers, which were executed by the rehabilitation robotic hand in a subject (a 28-year-old male)**.

During performance of passive movements of the right fingers, which were executed by the rehabilitation robotic hand, a significant increase of HbO and HbT values was observed for both the left SM1 and the left SMA (uncorrected, *p* < 0.01). In addition, a significant increase of HbT value was also observed for the left PMC and PFC during performance of passive movements of the right fingers. By contrast, in terms of HbR value, only the hand somatotopic area of the left SM1 showed a significant decrease, compared with other ROIs (uncorrected, *p* < 0.01) (Figure 2B). In the time-course of actual hemodynamic responses, increased HbO and decreased HbR values were observed in ROI on the SM1 during performance of passive movements of the right fingers, which were executed by the rehabilitation robotic hand, and decreased or increased during rest phases (Figure 3).

## Discussion

HbO, HbR and HbT, which measure neural activity indirectly by detecting hemodynamic changes of the underlying cerebral cortex, are the most commonly used parameters of fNIRS (Irani et al., 2007; Perrey, 2008). The rationale for use of these parameters is based on the concept that neural activation in response to a stimulus results in increased energy demands in the activated area; consequently, an increase of HbO and concomitant decrease of HbR in the activated area occurs during neural activation (Perrey, 2008; Leff et al., 2011). In the current study, we investigated change of HbO, HbR, and HbT in five ROIs (SM1, hand somatotopy of the SM1, PMC, SMA, and PFC) during performance of passive movements of the fingers, which were executed by the rehabilitation robotic hand. With regard to HbO and HbT values in each of the five ROIs, we observed cerebral activation in the hand somatotopy of the contralateral SM1 and total SM1, contralateral PMC and SMA and contralateral PFC. In terms of HbR value, we observed cerebral activation only in the hand somatotopy of the contralateral SM1. On the other hand, the hemodynamic responses in ROI on the SM1 showed irregular fluctuation during the rest phases, as shown in Figure 3. These results might be related to repetitive stimulation to the cortical area or effects of nonlinear neurovascular coupling (Hudetz et al., 1992; Mayhew et al., 1996; Toronov et al., 2000).

Our rehabilitation robotic hand appears to work for the brain mainly via proprioceptive input by passive movements of fingers. This proprioceptive input is the sense for the change of the relative position of the hand and strength of passive movements, and is generated from millions of sensory receptors in skin, muscles, joints, and ligaments. Since introduction of functional neuroimaging techniques, many studies have reported activation of both the primary motor cortex (M1) and primary somatosensory cortex (S1) as a result of passive movements (Mima et al., 1999; Radovanovic et al., 2002; Francis et al., 2009; Blatow et al., 2011; Lee et al., 2012). Recently, using fMRI, Szameitat et al. (2012) reported that passive movements of wrist joint induced cortical activation in the SM1and SMA, and this cortical activation pattern was similar to that of active movements of wrist joint (Szameitat et al., 2012). However, training with active movements induces more significant improvements in motor performance with facilitation of cortical networks, compared with passive movements (Lotze et al., 2003; Perez et al., 2004).

Therefore, we believe that our results indicating activation of the SM1 by the rehabilitation robotic hand are consistent with those reported in previous studies. The pathway of M1 activation by somatosensory stimulation has not been clearly elucidated. Previously, it was thought that an afferent input arrives at the M1 through the S1 (Forss et al., 1994). However, there is general agreement that the M1 receives somatosensory input directly from the thalamus or dorsal column (Desmedt and Cheron, 1980; Dinner et al., 1987; Canedo, 1997; Jang et al., 2012). In addition, results of animal studies have demonstrated direct involvement of somatosensory input to the motor cortex in execution of voluntary movements (Asanuma and Arissian, 1984; Favorov et al., 1988), and branching axons to dorsal column nuclei were observed in the corticospinal tract (Bentivoglio and Rustioni, 1986; Martinez et al., 1995; Steward et al., 2004). However, in the current study, except for the contralateral SM1, the secondary motor area, including contralateral SMA and contralateral PMC, were activated by execution of our rehabilitation robotic hand. The secondary motor area is known to receive somatosensory input directly and this appears to be the basic mechanism of activation of the secondary motor area; likewise, activation of the SM1 (Hummelsheim et al., 1988; Rouiller et al., 1999; Chung et al., 2005; Kishi et al., 2009).

Although many rehabilitation robots have been developed for patients with brain injury, studies on cortical activation during execution of the robots are limited (Aisen et al., 1997; Krebs et al., 1998; Volpe et al., 2000; Lum et al., 2002, 2006; Kahn et al., 2006; Masiero et al., 2007; Kwakkel et al., 2008; Rabadi et al., 2008; Housman et al., 2009; Lo et al., 2010; Conroy et al., 2011; Lapitskaya et al., 2011). To the best of our knowledge, only a few studies using transcranial magnetic stimulation or fNIRS have demonstrated the cerebral effect by rehabilitation robots (Blicher and Nielsen, 2009; Kamibayashi et al., 2009; Li et al., 2012). In 2009, using transcranial magnetic stimulation in 13 normal subjects, Blicher and Nielsen investigated the cortical effect of robotic gait training using a driven gait orthosis (Blicher and Nielsen, 2009). They found that the decrease in short-interval intracortical inhibition after passive training in this gait robot may reflect a decrease in intracortical GABA activity, which could aid in acquisition of new motor skills. During the same year, Kamibayashi et al. investigated change of corticospinal excitability to the lower limb muscles using transcranial magnetic stimulation and transcranial electrical stimulation of the motor cortex while 13 normal subjects stepped passively in a robotic driven-gait orthosis (Kamibayashi et al., 2009). According to their findings, corticospinal excitability to the lower limb muscle was facilitated by load-related afferent inputs. In a recent study, Li et al. (2012), who developed a motion-tracking training robot by elbow flexion-extension movements, demonstrated the effect of this robot using fNIRS in 14 normal subjects (Li et al., 2012). They reported an increase in motion tracking precision and cortical activation in motor-control-related regions (SM1, SMA, PMC, and somatosensory areas) following motion-tracking training. In addition, they observed that, in terms of cortical activation, bimanual training was better than single-limb training.

In conclusion, we investigated cortical activation patterns during execution of our rehabilitation robotic hand; according to our results, the contralateral SM1, along with the contralateral PMC, contralateral SMA, and contralateral PFC were activated. Our results appear to suggest that execution of the rehabilitation robotic hand could induce cortical activation; therefore, we believe that our results would be helpful in research on development of rehabilitation robots. In addition, fNIRS could be a useful tool in research on the cortical effect of rehabilitation robots. For the clinical application, conduct of further studies on the optimal conditions for cortical activation and robot-assisted rehabilitation therapy by decoding of movement intention will be necessary (Gomez-Rodriguez et al., 2011). In addition, further studies on the training effect of this robotic hand in normal subjects and the clinical effect for patients with brain injury are also encouraged. However, the limitation that we did not monitor passive movements using an electromyographic method should be considered.

## Conflict of interest statement

The authors declare that the research was conducted in the absence of any commercial or financial relationships that could be construed as a potential conflict of interest.

## Footnotes

^1^http://bisp.kaist.ac.kr/NIRS-SPM
